# Helicobacter pylori infection associated with an increased risk of colorectal adenomatous polyps in the Chinese population

**DOI:** 10.1186/s12876-018-0918-4

**Published:** 2019-01-21

**Authors:** ChangxiChen, Yushan Mao, Juan Du, Yimin Xu, Zhongwei Zhu, Hongbao Cao

**Affiliations:** 1 Department of Gastroenterology, Hospital of Zhenhai Refine-Chemical Company, 168 N Tianyi Rd, Zhenhai District, Ningbo, 315207 China; 2 Department of Endocrinology, the Affiliated Hospital of Ningbo University Medical College, Ningbo, 315020 China; 3 Statistical Genomics and Data Analysis Core, National Institute of Mental Health, National Institutes of Health, Bethesda, MD 20892 USA

**Keywords:** *Helicobacter pylori*, Colorectal adenomatous polyps, Colorectal carcinoma, Logistic regression, China

## Abstract

**Background:**

Gastric *Helicobacter pylori* (*H. pylori*) is linked with chronic gastritis, peptic ulcer disease, and gastric malignancy. This study aims to investigate the association of gastric *H. pylori* with colorectal adenomatous polyps (CAP) in the Chinese population.

**Methods:**

One thousand three hundred seventy five workers of China Petroleum and Chemical Corporation Sinopec Zhenhai Refining & Chemical Branch were recruited. Carbon-13 urea breathes test, and colorectal biopsies were utilized to detect *H. pylori* and CAP. The correlation between the number and distribution of CAP with *H. pylori* infection (HPI) was determined. Logistic regression models were applied to calculate the effect of *H. pylori* on the risk of CAP and pathway studio was used to attribute the cellular processes linking *HPI* and adenomatous polyps.

**Results:**

One hundred Eighty participants were diagnosed as CAP, and 1195 participants were classified as healthy control. The prevalence of *HPI* in the CAP group was significantly higher than that in the healthy control group (57.8% verse 40.1%) (*p*<0.001). It was the number not the distribution of CAP corrected with *H. pylori* status. An increased risk of CAP was found to be associated with *H. pylori* (OR = 3.237; 95.0% CI 2.184–4.798, *p* = 0.00) even after multiple parameters adjustment. Pathway studio analysis demonstrated that *HPI* connected with CAP at multi-level.

**Conclusions:**

*HPI* is associated with an increased risk of CAP in the Chinese population.

**Electronic supplementary material:**

The online version of this article (10.1186/s12876-018-0918-4) contains supplementary material, which is available to authorized users.

## Background

Although advanced treatment strategy has significantly declined the mortality, colorectal carcinoma (CRC) is estimated to be the 3rd malignancy and the 4th primary cancer-related deaths [1–3]. 20% of patients without any symptom at the time of diagnosis may present with contiguous invasion, transperitoneal spread, and lymphatic or hematogenous dissemination [4–6]. In the matter of geographic pattern, the prevalence has increased in Canada, Australia, and the United States, while India and China show a relatively low risk [4, 7, 8].

Colorectal adenomatous polyp (CAP) can be considered as precancerous lesions, for hyper-proliferative epithelial cells can transform from adenomatous stage to CRC stage [9, 10]. The early diagnosing and early treatment of CAP depends on colonoscopy, which could significantly decrease the incidence of CRC. It is even demonstrated that even one-time colonoscopic screen at the age of 55-year could reduce 30–50% mortality [11–13]. Nowadays, more strategies are sought to promote eligible individuals to take part in this CRC screening programs.

*Helicobacter pylori* (*H. pylori*) affects about 50% of the world population which can lead to chronic gastritis, peptic ulcer disease, and even gastric malignancy [14–19]. However, there is still a lack of evidence to confirm the existence and the extent of association between *H. pylori* and CAP. This research aims to investigate whether *HPI* could be used as an alternative to colonoscopy screen to associate with CAP and to quantify such risk in Chinese population.

## Methods and materials

### Participants’ selection

One thousand three hundred seventy five workers and staff members of China Petroleum and Chemical Corporation Sinopec Zhenhai Refining & Chemical Branch who underwent regular physical check-up and colonoscopy from March 2013 to October 2014 in Zhenhai Lianhua Hospital were recruited. This study protocol was approved by the Ethics Committee of Zhenhai Lianhua Hospital, and the written consents from participants were obtained. The number and distribution of CAP were determined. Participants with inflammatory bowel disease, CRC, suboptimal bowel preparations, uncompleted colonoscopies, and short of *H. pylori* information were excluded.

### Specimens

Encountered colorectal polyps were harvested and resected, which were further classified by the distribution as right-sided colon phenotype (cecum, ascending, and transverse colon), left-sided colon phenotype (descending colon, sigmoid, and rectum), and whole colon phenotype. All specimens were paraffin embedded.

### Carbon-13 urea breath test for *HPI*

A non-invasive carbon-13 urea breath test (^13^C UBT) kit ordered from Shenzhen Headway Bio-Sci & Tech Co., Ltd. (Shenzhen, China) was used to detect *H. pylori* according to the manufacturer’s instruction. In brief, participants drunk 50 ml commercial orange juice deliquated with ^13^C-urea (75 mg). Expelled air samples were collected into Tedlar gas bags before and 30-min after the tracer ingestion, which was further detected with mass spectrometer systems. The cutoff value of delta over baseline (DOB) was set up as 4.0 per thousand to diagnose as *H. pylori* positive.

### Pathway analysis

To find the association between *HPI* and CAP, the shortest path algorithm in pathway studio [20] was employed with default parameters, which built connections with the cell, cell processes, compounds, organs, and functional classes. Each edge was annotated with text mining result which was supported by one or more references, and the detailed information could be identified in Additional file 1, which is also online available at http://gousinfo.com/database/Data_Genetic/HPI_AP_Suplementary.xlsx, including the titles and sentences where a relationship has been recognized.

### Statistical analysis

The distributions of continuous and categorical variables were analyzed with Student’s *t*-test and Chi-square test. A multivariable logistic regression analysis was adopted to estimate the predictive effect of *H. pylori* on the risk of CAP. *P* < 0.05 was considered as statistical significance.

## Results

### Population and clinicopathological characteristics

Characteristics of the participants were outlined in Table 1. The population was composed of 1375 subjects, among which 180 subjects were diagnosed as CAP by colorectal biopsies, and the other 1195 subjects were classified as healthy control. The average age of participants was 53.7 ± 10.5 for healthy control subjects and 58.6 ± 11.3 for subjects with CAP. The ratio of male gender among *H. pylori* positive participants (147/880, 16.7%) was significantly higher than that in female (33/495, 6.7%) (χ^2^ = 28.057,*p*<0.001). Participants with CAP showed a considerably increased age, waist circumference (WC), body mass index (BMI), systolic blood pressure (SBP), fasting blood glucose (FBG), uric acid (UA), and ^13^C DOB compared with healthy control.

**Table 1:** Distribution of clinical variables

	Healthy control(*n* = 1195)	CAP group (*n* = 180)	*t*	*p*
AGE (year)	53.7 ± 10.5	58.6 ± 11.3	5.811	< 0.001
WC (cm)	82.6 ± 8.8	85.5 ± 8.6	5.553	< 0.001
BMI(kg/m2)	23.6 ± 2.8	24.3 ± 2.9	3.077	=0.002
SBP(mmHg)	123(113,134)	126 (118,138)	3.270*	=0.001
DBP(mmHg)	78 (72,85)	79 (72,86)	0.763*	=0.445
TC(mmol/L)	4.87(4.34,5.49)	5.00(4.35,5.63)	1.123*	=0.261
TG(mmol/L)	1.20(0.82,1.72)	1.32(0.92,1.81)	1.703*	=0.089
HDL-C(mmol/L)	1.53(1.31,1.77)	1.45(1.28,1.74)	1.531*	=0.126
LDL-C(mmol/L)	2.64(2.17,3.16)	2.81(2.33,3.25)	1.904*	=0.057
FBG(mmol/L)	5.07(4.73,5.49)	5.14 (4.85,5.67)	2.713*	=0.007
UA(μmol/L)	321(265,380)	328(275,397)	1.707*	=0.088
^13^C(DOB)	1.80(0.60,11.7)	5.95(1.00,15.7)	3.601*	< 0.001

note:* = Z value

### The prevalence of *HPI* in CAP

The prevalence of *HPI* in the CAP group (104/180, 57.8%) was significantly higher than that in the healthy control group (479/1195, 40.1%) (χ^2^ = 20.1,*p*<0.001). CAP was mainly distributed in the left colon (58.3%). After Chi-square test, *H. pylori* prevalence in multiple polyp patients was higher than solitary polyp patients (66.2% vs. 51.5%) (χ^2^ = 3.944,*p* = 0.047), while patients with different polyp location phenotypes showed no significant difference of *H. pylori* prevalence (χ^2^ = 0.597,*p* = 0.742, Table 2).

**Table 2:** The relation of CAP distribution and location with *HPI*

		*H.Pylori* positive (*n* = 104)	*H. pylori* negative (*n* = 76)	*p*
Number	solitary	53	50	0.047
	multiple	51	26	
Location	left-sided colon	59	46	0.742
	right-sided colon	27	20	
	whole colon	18	10	

### *HPI* associated with a higher odds ratio of CAP

Multiple logistic regression models for *HPI* concerning colorectal adenomas polyps were performed. Without parameters adjusted (model 1), *HPI* had a higher odds ratio (OR) of CAP (OR = 2.863;95.0% CI 1.975–4.150,*p* = 0.000). After adjustment of age and gender (model 2), subjects with *H. pylori* showed more prevalence of CAD (OR = 3.104;95.0% CI 2.104–4.775,*p* = 0.000). After additional adjustment for WC, BMI, systolic blood pressure (SBP), diastolic blood pressure (DBP), total cholesterol (TC), triglyceride (TG), LDL, and HDL (model 3), the results did not materially change (OR = 3.237;95.0% CI 2.184–4.798,*p* = 0.000) (Table 3). All of these data indicated that *HPI* could be used as an independent factor in the risk analysis of CAP.

**Table 3:** Multiple logistic regression models for *H.pylori* concerning colorectal adenomas polyps

*H. pylori*	Partial regression coefficient	Standard error	Waldχ^2^	*p*	OR (95.0% CI)
Model 1	1.052	0.189	30.821	0.000	2.863 (1.975,4.150)
Model 2	1.122	0.198	32.651	0.000	3.104 (2.104,4.577)
Model 3	1.175	0.201	34.213	0.000	3.237 (2.184,4.798)

Note: no parameter was adjusted in Model 1; Model 2 adjusted for age, gender; Model 3 adjusted for age, gender, WC, BMI, systolic and diastolic blood pressure, total cholesterol, triglyceride, LDL, HDL

### Potential functional pathways connecting CAP and *HPI*

**Fig. 1:**
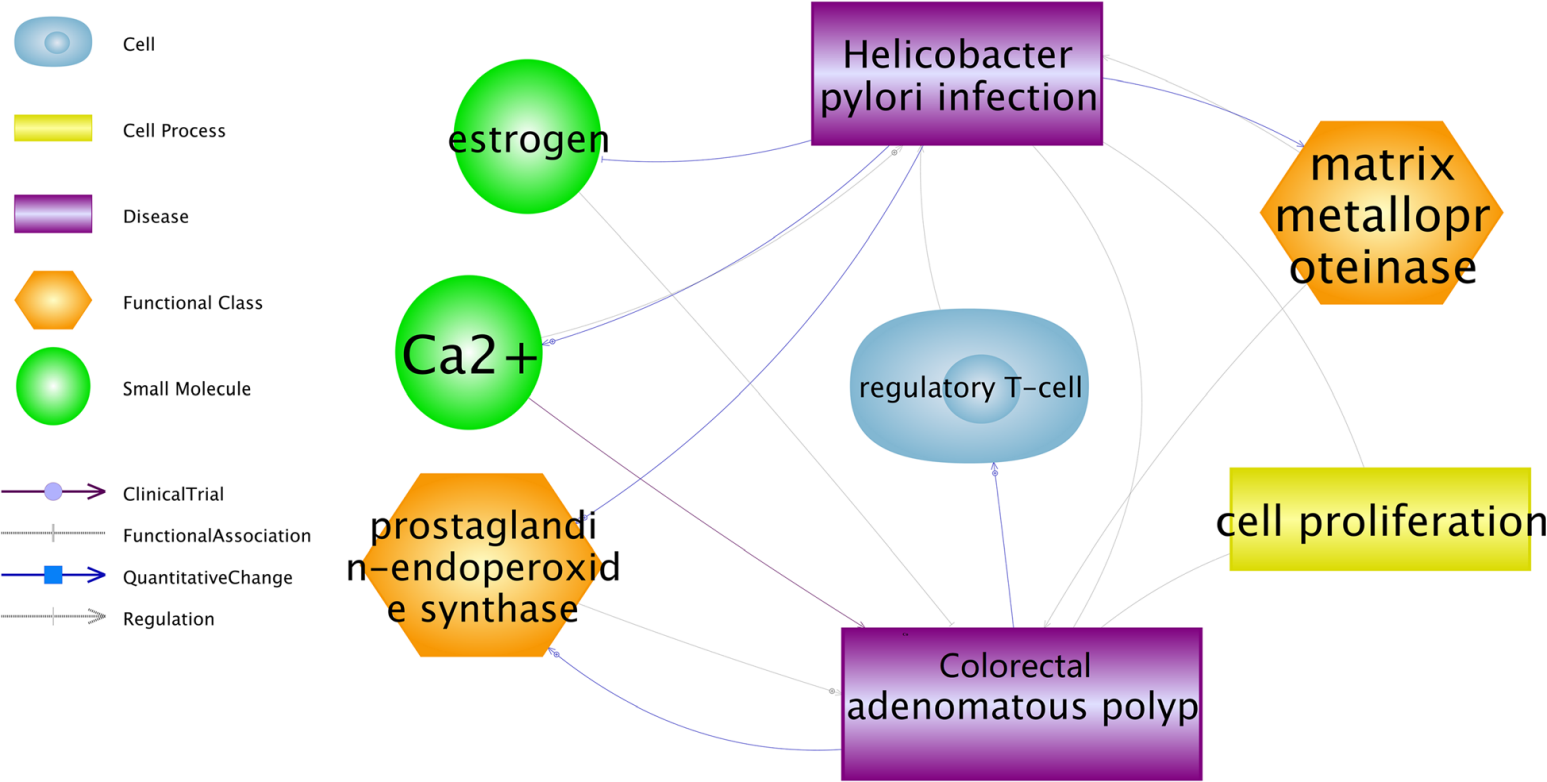
Pathway analysis showed multiple cellular processes related to *H. pylori* infection and colorectal adenomatous polyps. A biological process annotation was created using the Pathway Studio 7.0 program to identify cell process and functional class associated with *H. pylori* infection and colorectal adenomatous polyps and the most common cellular processes were matrix metalloproteinase, prostaglandin-endoperoxide synthase, and mucin

*HPI* demonstrated possible multi-level connections with CAP as shown in Fig. 1. The most common cellular processes were matrix metalloproteinase, prostaglandin-endoperoxide synthase, and cell proliferation. All of these might be the transformation processes involved in *Helicobacter pylori* infection and further development of adenomatous polyp. To note, for each relationship (edge) presented in Fig. 1, there were one or more supporting references, which have been provided in the Additional file 1 available at http://gousinfo.com/database/Data_Genetic/HPI_AP_Suplementary.xlsx.

## Discussion

*HPI* shows clearly an association with the development of gastric carcinoma, while both positive [21–23] and negative [24, 25] associations reported with colorectal neoplasia. This investigation finds that *H. pylori* prevalence in CAP group is significantly increased than that in the healthy control group. *H. pylori* prevalence in multiple CAP group is higher than that in single CAP group. Moreover, pathway analysis revealed multiple pathways through which *HPI* could promote the development of CAP (Fig. 1). All of these indicate that *HPI* is associated with an increased risk of CAP and bioinformatics analysis suggests that functional processes of matrix metalloproteinase, prostaglandin-endoperoxide synthase, and mucin may link the pathogenesis of *HPI* and CAP.

The literature-based functional pathway analysis revealed five forward pathways (HPI➔CAP) and one backward pathway (CAP➔HPI), as shown in Fig. 1. These pathways could help to understand the potential mechanisms that HPI may influence the pathology of CAP. Specifically, HPI could up-regulate the expression of matrix metalloproteinases [26], which may take part in not only colorectal carcinogenesis from adenomatous polyps but also colorectal tumor invasion and initiation of metastatic cascade [27]. HPI has been known to induce the expression of pro-inflammatory cyclooxygenase enzyme (COX-2) [28], and cyclooxygenase has been suggested as a promoter for CAP in familial adenomatous polyposis [29]. Additionally, HPI has been shown to decrease the level of estrogen [30] that protect against the development of colorectal cancers and adenomatous polyps [31]. HPI could also increase Ca2+ concentration [32], and Ca2+ is a critical chemopreventive agent in adenomatous polyps after polypectomy and after colorectal surgery for colorectal cancer. [33] On the other, pathway analysis also revealed a potential CAP➔HPI pathway. The underlying literature information showed that human adenomatous polyps are accompanied with accumulated Treg, [34] and Tregs contribute to the persistence of HPI [35]. These forward and backward pathways suggested a possible vicious circle where HPI associates with CAP in a promoting manner.

*HPI* is detected using a non-invasive carbon-13 urea breath test, and CAP is diagnosed by colonoscopy, all of these suggest the reliable of the results. Confounding elements such as age, gender, and metabolism related parameters cannot be neglected. The participants involved in this study are age and gender-matched, and multiple logistic regression models adjusted for multiple parameters shows *H. pylori* could be a risk factor for CAP. Patients with two or more colorectal adenomatous polyps are a high-risk group for *HPI* and the development of advanced adenomas cancer [36].

Based on previous researches, *HPI* probably occurs in childhood or adolescence and is associated with of increased gastrin release, which could promote the development of potential adenocarcinoma. Patients who have undergone colonoscopic surveillance and have colorectal adenomatous polyps removed show low chance of CRC development in the future. For most colon cancers are considered to have a premalignant adenomatous polyp phase [37, 38], it is important to perform colonoscopic surveillance, while in some cases such an operation is not practicable owing to the concern of complication and the lack of advanced medical intervention.

A positive relation between *H. pylori* and colorectal polyps is observed, which is in accordance with the CRC mouse models [39] and other population investigation [23, 40–42]. Moreover, for the first time, our data show that *HPI* could be used as an independent factor in the risk analysis of CAP in China. The pathogenic mechanisms responsible for this association remain uncertain. *H. pylori* have been detected in colorectal malignant tissues; however, the possibility that *H. pylori* are a direct activator of colonic carcinogenesis remains purely hypothetical. On the other hand, experimental data have suggested some potential oncogenic interactions between *H. pylori* and colorectal mucosae, such as inflammatory responses induction and perpetuation, gut microflora alteration, and gastrin release [43]. Because our study is based on a regular physical check-up instead of an epidemiology-based investigation, it may have some limitations to interpret the results. More prospective, long-term epidemiology-based investigations are urgent to confirm the link between *H. pylori* and CAP, which can be used as an alternative to colonoscopy.

## Conclusion

This study supports a possible connection between *HPI* and the risk of CAP which may require further investigation.

## Additional file

Additional file 1: Supporting information for the pathway analysis. Additional file 1 presents the detailed information of each and all relationships identified from the pathway analysis (Fig. 1), including relationship type and the supporting references for each relationship (title, publication year, author and related sentences).(XLSX 22 kb)

## Abbreviations

BMI: Body mass index
CAP: Colorectal adenomatous polyps
DBP: Diastolic blood pressure
FBG: Fasting blood glucose
*H. pylori*: *Helicobacter pylori*
OR: Odd Ratio
SBP: Systolic blood pressure
TC: Total cholesterol
TG: Triglyceride
UA: Uric acid
WC: Waist circumference
